# Wireless sensor network-based health monitoring and smart home assistance for the older adults in sports and wellness towns

**DOI:** 10.3389/fpubh.2024.1399648

**Published:** 2024-10-23

**Authors:** Dapeng Yang, Junqi Wang, Li Huang, Tongling Wang

**Affiliations:** ^1^College of Physical Education, Huainan Normal University, Huainan, China; ^2^School of Physical Education and Sport, Henan University, Kaifeng, China; ^3^Conservatory of Music, Huainan Normal University, Huainan, China; ^4^Institute of Physical Education, Huzhou University, Huzhou, Zhejiang, China

**Keywords:** artificial intelligence, rural revitalization, sports and wellness town, k-means algorithm, healthy aging through

## Abstract

**Background:**

Integrating technology with rural development is essential for addressing the unique challenges faced by aging populations in rural areas. China’s national rural revitalization strategy emphasizes the importance of developing characteristic towns that focus on health and wellness demands, particularly for the older adults. However, there is a gap in the literature regarding the systematic use of technology to support the health and daily living needs of this demographic.

**Objective:**

This study aims to bridge this gap by proposing a wireless sensor network (WSN)-based system that integrates health monitoring and smart home assistance, specifically tailored for older adults residents in sports and wellness towns.

**Methods:**

The system’s design involves collecting and analyzing historical activity data and basic physiological parameters from older adults residents’ homes. The data are cleaned, combined and stored to prepare it for behavior analysis, which is vital for controlling smart home equipment. Preliminary health assessments are conducted using the collected physiological data. A hybrid network leveraging Sub-G and Wi-Fi technologies is optimized for the purposes of collecting and uploading data, based on a comparative analysis of wireless communication options.

**Significance:**

This study significantly contributes to the advancement of sports and wellness towns by promoting healthy aging through cutting-edge technological solutions.

## Introduction

In China, the challenges of rural development are of paramount importance (1) and require innovative solutions that differ from urban development strategies (2). The country has recognized the need for novel approaches to stimulate rural revitalization (3) and establish a sustainable economic and social framework in these areas (4). As a result, rural tourism has been identified as a key driver of innovation and business growth in the countryside (5). To support this development, various rural regions across China have implemented tailored policies aimed at promoting tourism by capitalizing on the unique characteristics of each region (6).

In recent years, the concept of characteristic towns has gained traction nationwide, driven by national policies aimed at economic and social industrial classification (7). The earliest successful example of this type of town is Yunqi Town in Hangzhou, Zhejiang Province (8). Academic studies on characteristic towns emphasize the need to understand their development characteristics and establish them through precise governance (9).

Developing characteristic towns under the Rural Revitalization Strategy leverages the unique features of small towns to achieve large-scale development, increase farmers’ income, and create strategic opportunities for these towns (10). The foundation of characteristic towns should be improving people’s livelihood, utilizing natural and cultural resources, and promoting the healthy development of small-town economies (11).

In line with the Healthy China strategy, the integration of sports and healthcare is being explored as a means to promote a symbiotic relationship that can lead to the formation of positive synergies (12). This integration is particularly relevant to the development of sports and wellness towns, where health services are not solely reliant on medical interventions but are also supported by sports and leisure activities.

In the context of sports and wellness towns, the use of a wireless sensor network (WSN)-based system for health monitoring and smart home assistance for the older adults becomes particularly relevant. The WSN system is proposed as a solution to efficiently manage the health and wellness of the older adults population, ensuring their safety and comfort within these towns. The system’s capability to collect and analyze real-time health data allows for the early detection of health issues and the provision of timely medical assistance, thereby enhancing the quality of life for the older adults and contributing to the overall development of characteristic towns. Furthermore, the smart home assistance system is designed to support the older adults in their day-to-day activities, promoting independent living and reducing the need for constant supervision.

In order to support the development of characteristic towns and promote rural revitalization, this study proposed a wireless sensor network (WSN) system for health monitoring and smart home assistance of the older adults in sports health towns. The motivation for using a WSN system includes its ability to collect and analyze large volumes of data, improve the efficiency of health monitoring, and enhance the quality of life for the older adults. The smart home assistance system is designed to provide older adults residents with greater control over their home environment, ensuring their safety and well-being. The health monitoring system collects historical activity data and physiological parameters from the older adults’ homes, which are then cleaned, fused, and analyzed. These data help make preliminary health assessments and support the older adults in managing their daily activities.

## State of the art

### Overview of characteristic wellness towns and rural revitalization

As the name suggests, a characteristic wellness town is a distinctive community focused on wellness (13). The town boasts unique natural scenery and a distinct internal theme, highlighting is the importance of leveraging its excellent natural resources to develop rural tourism. Furthermore, characteristic tourist towns should also comprehensively manage the current towns from the perspectives of commerce, agriculture, and pension sectors, transforming them into prime destinations for leisure. This characteristic tourist town offers a relaxed lifestyle, a vibrant artistic culture, an exquisite ecological environment, and a rich historical and cultural heritage (14). Upon meticulous analysis, it becomes evident that characteristic towns manifest in various forms, exemplified by the Ronda bullfighting town in Spain, Baishishan Hot Spring, and Chengdu Anren Town, among others. However, characteristic towns differ from general towns and often lack certain necessary functions, such as radiative, driving, and gathering capabilities (15).

It is worth noting that while the healthcare town primarily addresses the urgent healthcare needs of the older adults and sub-healthy individuals, it must not overlook the healthcare requirements of other age groups (16). The healthcare town should cater to all age groups, including middle-aged and older adults groups, pregnant women, infants and young children, and adolescents. Additionally, since the healthcare town is not merely a tourist attraction or a retirement town, it cannot lose its original residential attributes. The healthcare town should display as many local customs and cultural characteristics as possible and become a livable, nurturing, and attractive place for both tourism and permanent residence.

The rural revitalization strategy is a key initiative pioneered by China in response to the actual conditions within the country, and it has become the fundamental program guiding the economic development of rural areas for the next three decades. The strategy aims to advance the modernization of agriculture, reduce the gap between urban and rural areas, and foster ecological civilization and agricultural development. By effectively addressing the issues of agriculture, rural areas, and farmers, collectively known as the “three rural issues,” the strategy not only focuses on improving the quality of life for farmers but also ensures the stability of people’s livelihoods in the country. In recent years, with the rise of the rural revitalization strategy, the Chinese government has been unwavering in its commitment to reform and innovation, always adhering to the concept of comprehensive development to promote the integration of urban and rural areas. The implementation of this strategy reflects the Chinese government’s emphasis on rural development and its determination to achieve economic prosperity, social progress, and environmental sustainability in rural areas through a strategy of integrated development (17).

### Integration of sports and tourism

This study is based on the definition of sports tourism as a type of social and cultural activity that possesses attributes of both sports and tourism, arising from individuals traveling for specific sports purposes or engaging in sports-related activities during their travels. This article defines the “integration of sports and tourism with healthcare” as combining the concepts of sports health preservation, ecological resources and tourism activities so that people can participate in sports activities while traveling and leisurely to experience a healthy lifestyle (18). Tourism consumption and sports consumption mutually support each other. Some elements of sports are integrated into tourism to address their own problems, leading to value addition. Tourism needs an international market and relies on events for marketing. Therefore, tourism attracts tourists and enhances the value of the tourism industry through the huge influence of sports events. The sports industry also has strong penetration and relevance, with a large number of fans and rich experience in organizing events. The stronger the correlation between industries, the higher the efficiency of mutual utilization of resources. Tourism and sports have strong relevance and similarity in terms of physical fitness and social communication. Tourism provides a better platform for the sports industry, which provides a wider demand and profit space for tourism (19).

The document *Sports* clearly states that “Sports and leisure characteristic towns are in the process assisting the construction of a new type of urbanization and a healthy China and promoting poverty alleviation. Work, a space area integrated with the theme of sports and leisure, a national fitness development platform and sports industry Base.” This article adopts Mei Li’s definition: a sports and leisure town is one where the sports industry is the core, combined with a characteristic sports culture, aiming to enhance physical health while integrating sports, leisure, tourism, health, and entertainment into a multifunctional space.

### The relationship between rural revitalization and characteristic towns

Characteristic towns are often spatially close to the countryside. Economically, the core industry agglomeration function of the town will have a radiating effect on the countryside. The related industrial processing, talent training and employment services between the town and the rural structure will better promote the rural area. At the same time, the green, healthy, comfortable and livable space environment created by the characteristic town, surrounded by abundant resources and location advantages such as education, medical care, and culture, can fundamentally promote the endogenous development of the countryside and promote the local Prosperity of industries is the foundation and prerequisite for rural revitalization. Characteristic towns are the key to urban–rural integration and an important node connecting strategy; they integrate urban and rural functions within a reasonable spatial range and are in line with the “prosperous industry, ecological livability, and rural style.” Civilized and prosperous” new villages are connected to form an integrated development pattern of urban and rural areas, thereby driving and accelerating the pace of rural revitalization (20).

The construction and development of healthcare towns promote local, improve regional competitiveness, and drive industrial development. Presently, the nation is confronted with both opportunities and challenges in the development of healthcare towns. On the one hand, the state has successively issued a series of healthcare policies, bringing huge opportunities for the development of healthcare towns.

**Figure 1:**
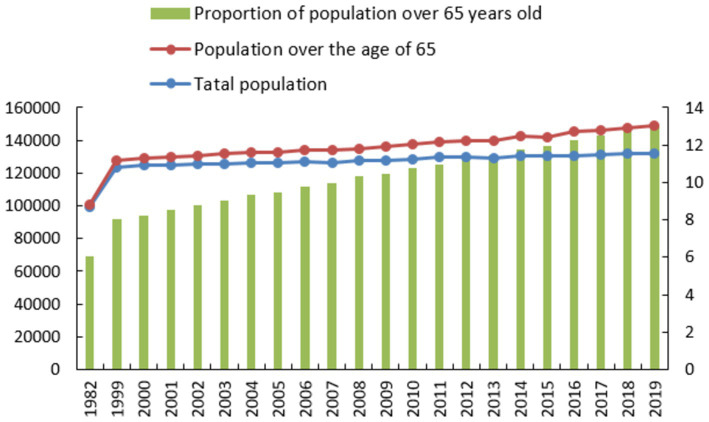
Proportion of the older adults over 65 years of age in the nation.

It mainly includes four aspects of policy output: national and local government subsidy policies for older adults care buildings and services, policy support for tourism investment and consumption, policy support for planning and construction of local health industry demonstration zones, and policies for characteristic town support. In such an environment, the healthcare town can have certain development advantages. However, China has not yet formed a healthy and complete healthcare industry system. There are still the following problems in the construction and development of healthcare towns: (1) The problem of homogenization of product content in healthcare towns is serious. At present, the construction of domestic healthcare towns is in full swing. However, due to the lack of communication between different regions or the arbitrary decision-making of some governments, the failure to adapt to local conditions has led to the increasingly serious situation of repeated construction of industrial projects, homogeneous competition, and the characteristics of industries and resources. Indistinct and indistinguishable. (2) Policies and regulations are relatively lagging behind. The healthcare industry’s development in the nation is still in the early stages, despite new policies and expanding projects, indicating significant potential for future improvement. Regulations and measures need to be improved in various ways to ensure that the development of the healthcare industry is in line with the actual situation. (3) The product model is relatively simple. The planning and design of healthcare towns by some companies is just simple, streamlined production. The basic healthcare industries are similar in type. There is a lack of innovation in health, healthcare, and older adults care services, and sustainable development is difficult. Kangyang Town should not only make full use of its own and external advantages but also fully tap its own characteristics (21). Starting from the actual situation of local social and economic development, centered on and giving full play to its own resource advantages and characteristics, creating characteristic healthcare services, constructing a relatively complete supporting service and industrial chain system, and improving core attractiveness. Second, on the basis of its own advantages, taking regional cultural characteristics as the carrier, adapting measures to local conditions, combining medical care and healthcare, realizing industrial upgrading and integration of production and city, creating a healthcare town with local characteristics, and promoting the prosperity of local industries. F1 shows the country when investigating sports and healthcare towns (Figure 1).

## Materials and methods

### Smart home health monitoring

This study utilizes data mining techniques to fully investigate the potential value of historical operation data from users’ daily lives, to mine the hidden user habits, and to develop a smarter home system. The implementation details are as follows: (1) Data collection: Sensors are deployed in the homes of the older adults to collect data on their physiological parameters and daily activities. These sensors form a network that continuously monitors vital signs such as heart rate, blood pressure, and movement patterns. (2) Data Processing: The collected data are cleaned, fused and stored for analysis. This includes preprocessing steps to remove noise and irrelevant information, ensuring that the data used for health monitoring is accurate and reliable. (3) Behavioral analysis: By analyzing activity data, the system can identify unusual patterns or behaviors that may indicate health problems. For example, a sudden decrease in physical activity or irregularities in vital signs may trigger an alert for further medical evaluation. (4) Outcome Reflections: The outcome section provides data and analyses of the system’s implementation, highlighting how effectively the system monitors the health status of older residents.

Cluster analysis involves selecting an appropriate algorithm to analyze and calculate a certain dataset and categorizing it based on the principle of minimizing intra-class distances and maximizing inter-class distances. It is also an unsupervised learning task. In the context of smart home systems, cluster analysis can be employed to identify habitual patterns with distinct personal characteristics in the home activities of the older adults, and to formulate intelligent control strategies for the smart home system (22).

Association analysis is used to discover and describe patterns of strongly correlated features in data and is often referred to as “sequence mining,” “activity monitoring,” and so on. The support calculation in association rules is shown in Equation 1:


(1) $$S\left(I\right)=\frac{\partial_{i}}{\left|D\right|}*100\%$$

If $S\left(I\right)$ itemsets whose intersection is empty, and *J* is the conclusion, then *I*= > *J* is called an association rule, and its support *S*(*I*= = > *J*) is shown by Equation 2:


(2) $$S\left(I\Rightarrow J\right)=S\left(I\cup J\right)$$

Then, the confidence *C*(*I*= > *J*) of this association rule is shown in Equation 3:


(3) $$C\left(I\Rightarrow J\right)=\frac{S\left(I\cup J\right)}{s\left(I\right)}$$

In a wellness house, the process of acquiring the behavior patterns of the older adults involves identifying their interests and frequent activities. The association rule algorithm helps the system discover these behavior patterns and provide intelligent services tailored to the older adults.

During the data collection process, sensors are needed. The wireless sensor is generally composed of a perception module and information. A large number of sensors are deployed in residences and are self-organized to form a network. The data transmission process is that the monitoring node transmits hop by hop along other nodes, then routes to the sink node after multiple hops, and finally reaches the management node.

**Figure 2:**
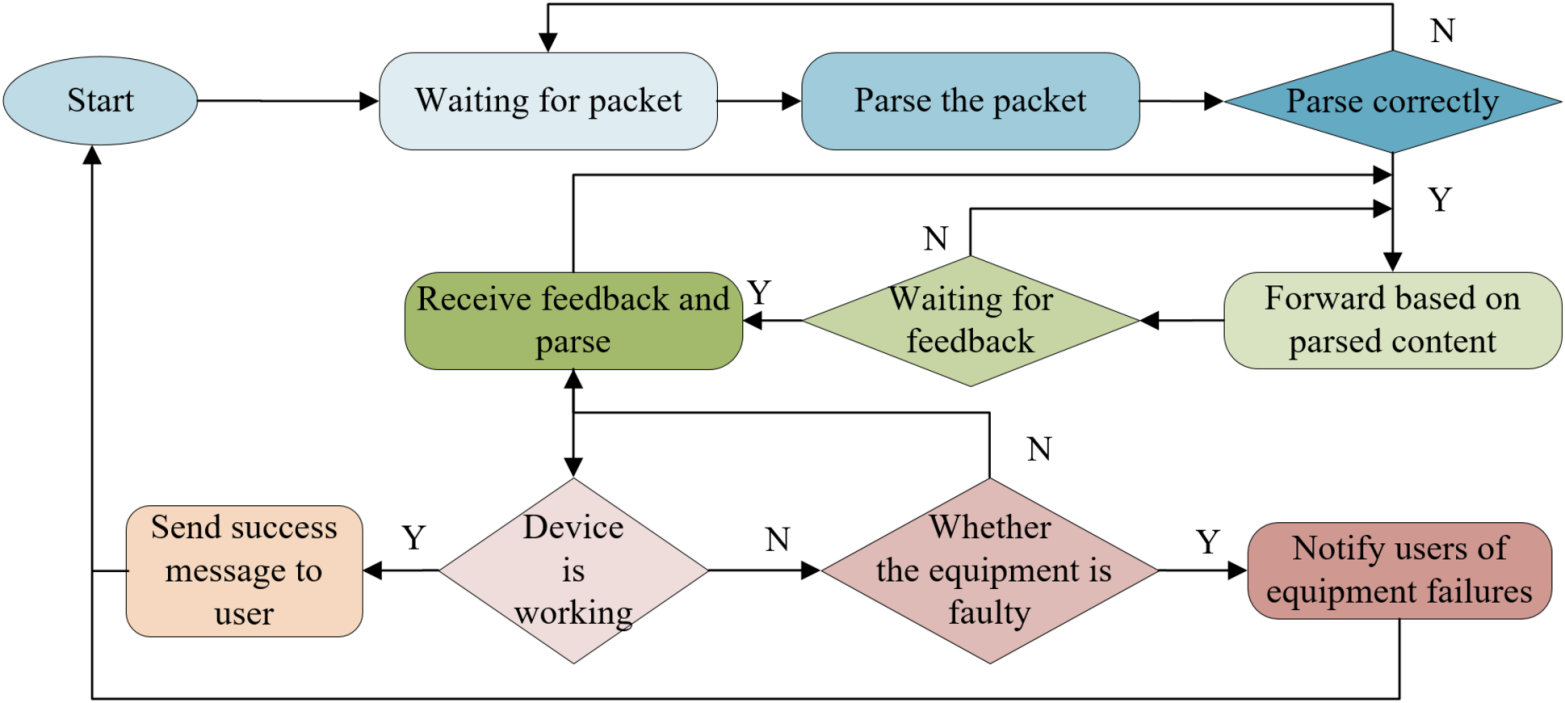
Workflow of the gateway.

For such smart devices, the gateway control module is the key. The SN8F5708 chip is selected as the main one to realize the function of the gateway, receive the control signal of the system through the serial communication mode, and send the data of the acquisition network. The gateway will parse the data packets sent by the server according to the specified protocol and then perform corresponding control. At the same time, the server can also receive the status information of the sensor. Taking the status of the gateway to be initialized as an example, the connection process is shown in Figure 2. It shows the step-by-step process that the gateway undergoes, starting from initialization, waiting for packets, parsing the packet correctly, and then either forwarding based on parsed content or notifying users of equipment status and potential failures.

### Demand analysis of multi-sensor data fusion

**Figure 3:**
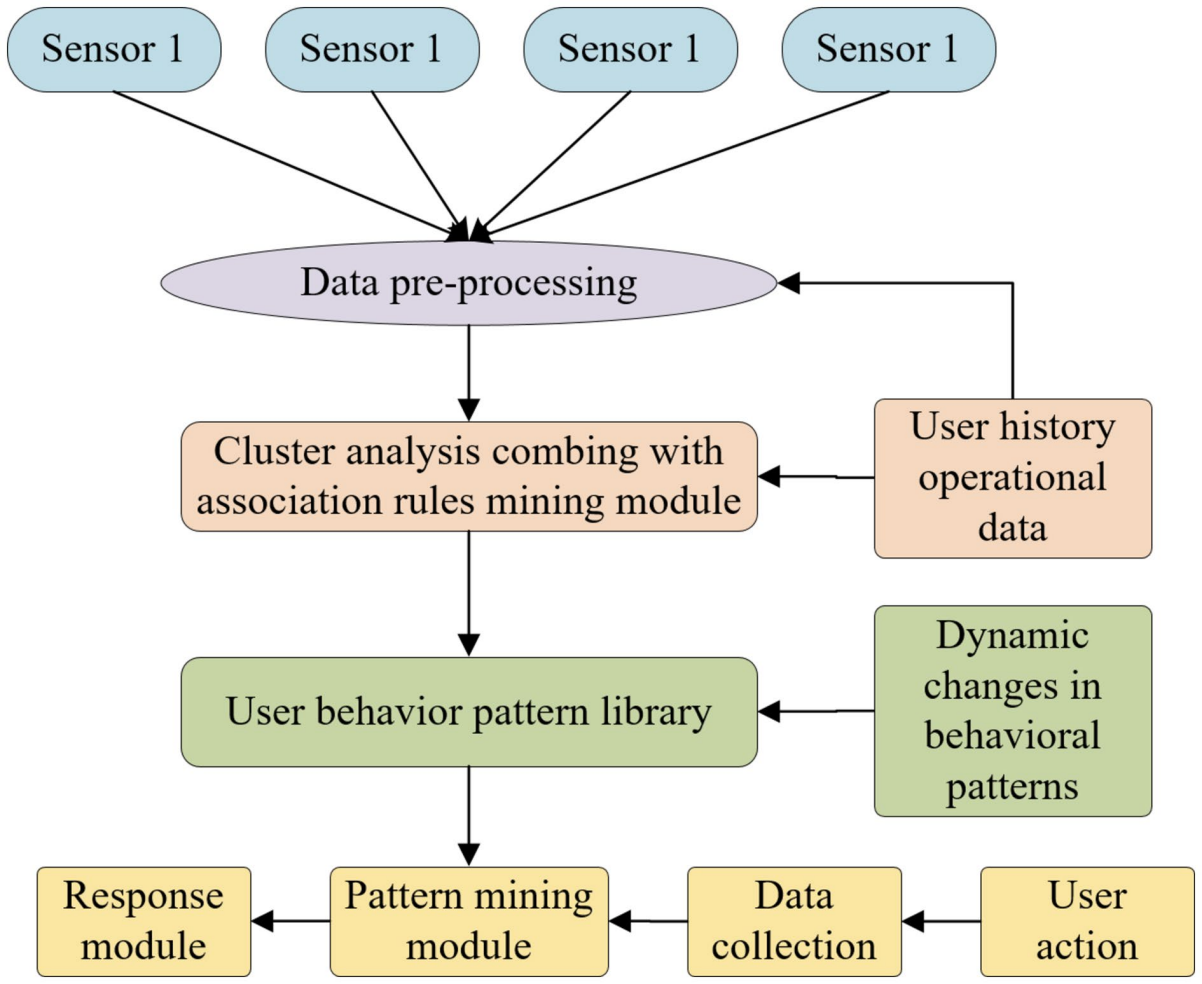
Structure diagram of multi-sensor data fusion and pattern analysis.

The main reason for using the principle of multi-sensor data fusion involves making full use of advantages, avoiding disadvantages, and improving the safety factor of the whole vehicle through redundant design. The functions realized by a multi-sensor fusion system are far more than the sum of the functions realized by these independent systems. The use of different sensor types can provide additional redundancy under environmental conditions where all sensors fail (23). The principle of multi-sensor data fusion is as follows: (1) multiple different types of sensors (active or passive) collect data on observation targets. (2) Transform the output data of the sensor (discrete or continuous time function data, output vector, imaging data or a direct attribute description) for feature extraction to extract a feature vector Yi representing the observation data. (3) Carry out pattern recognition processing on the feature vector Yi (such as clustering algorithms, adaptive neural network or other statistical pattern recognition methods that can transform the feature vector Yi into target attribute judgment, and so on), and complete the description of each sensor about the target. (4) The description data of each sensor on the target is grouped according to the same target, that is, associated. (5) The fusion algorithm is used to synthesize the sensor data of the target to obtain a consistent interpretation and description of the target. Multi-sensors produce data fusion, including useful data and useless data. Multi-sensor data combined with algorithm processing can eliminate data unrelated to current activities and at the same time use data of different times and spaces to make an explanation for a certain behavior data or description in order to carry out the classification of the behavioral activities of the older adults in sports and wellness towns throughout the network. The sensor data fusion process and pattern analysis structure are shown in Figure 3, which is divided into sensor data collection, data preprocessing and classification and identification.

The data collected by wireless sensors cannot be used for behavioral analysis immediately, and some methods need to be preprocessed. Conventional preprocessing of data includes data cleaning, data transformation, data reduction, and so on. The cosine similarity measure method is used to calculate the similarity between the two vectors, and its value range is [0, 1], shown in Equation 4:


(4) $$\cos\left(i_{1},i_{2}\right)=\frac{\sum_{z=1}^{t}i_{xz}i_{yz}}{\sqrt{\sum_{z=1}^{t}{\left(i_{xz}\right)}^{2}\sum_{z=1}^{t}{\left(i_{yz}\right)}^{2}}}$$

To supplement the missing values, the Newton interpolation method can be used, and it is shown in Equation 5.


(5) $$T_{t}\left(I\right)=f\left(i_{0}\right)+f\left[i_{0},i_{1}\right]\left(i-i_{0}\right)+f\left(i_{0},i_{1},i_{2}\right)\left(i-i_{0}\right)\left(i-i_{1}\right)$$

Noise is also called outlier or outlier data, so the nearest neighbor substitution method can be used to process noise data, and the data of this point is set to the value of the data point whose distance is the one, shown in Equation 6:


(6) $$\mathrm{Dist}\left(i_{x},i_{y}\right)=\sqrt{\overset{t}{\underset{z=1}{\sum }}{\left(i_{xz}-i_{yz}\right)}^{2}}$$

The collection of user behavior data mainly includes regional location, device name, transmission time, device status value, and so on. The data transformation involves converting the above values into which characters may appear. For example, the two states of “on” and “off” can use the values 1 and 0 to represent their corresponding states. The switch status and gear positions “high,” “medium,” and “low” can be expressed as 3, 2, and 1, respectively, using continuous numerical values. Additionally, numerical values like 0, 1, 2, 3, and 4 can be assigned to different intervals, such as bedroom, study, living room, and kitchen.

### K-means algorithm fused with SOM

The main motivation for using the k-means algorithm in this study is its effectiveness in handling large-scale datasets, such as historical activity data and physiological parameters of older adults residents collected from smart homes. K-Means is computationally efficient and can handle the multidimensional nature of the data efficiently, making it ideal for real-time monitoring and analyzing applications. It scales well with large datasets, which is critical for real-time health monitoring systems. The linear time complexity of the algorithm allows it to process large amounts of data quickly, ensuring timely insights and interventions. The SOM algorithm can maximize the guarantee that the training will not fall into the local optimal solution, at the cost of a long training time, while the k-means algorithm has a short clustering time but an optimal solution. Combining the advantages of SOM, the data can be input into the SOM network for initial clustering, and then these results can be used as the initial clustering centers of the k-means algorithm to obtain the final clustering results.

Randomly initialize the reference weight vector w for each neuron; (*y* = 1,2....*u*.), set the number of iterations *T*, set the initial learning rate no = 0.5, and its decreasing function is as follows Equation 7:


(7) $$\eta \left(t\right)=\eta_{0}\left(1-\frac{t}{T}\right)$$

where *t* is the current number of iterations.

Select the winning neuron. That is to say, the reference weight vector corresponding to all neurons is the smallest distance, which is the winning neuron. Euclidean distance of two m-dimensional category vectors $I_{x}$ , $I_{y}$ is as follows Equation 8:


(8) $$d\left(I_{x},I_{y}\right)=I_{x}-I_{y}=\sqrt{\overset{T}{\underset{w=1}{\sum }}{\left(I_{xw}-I_{yw}\right)}^{2}}$$

The distance between the winner weight vector in $M_{y}$ and the input sample *I* is as follows Equation 9:


(9) $$d_{x,y}=\min I_{x}-I_{y}$$

Expanding Equation 10, the resulting expression is as follows:


(10) $$I-M_{y}=\sqrt{{\left(I-M_{y}\right)}^{r}\left(I-M_{y}\right)}$$

From Equation 11, it can be known that the Euclidean distance between the two unit vectors is the smallest, and $M_{y}^{r}I$ is the largest.


(11) $$M_{y}^{r}I=\max_{\left(r\cdot 1,2\ldots ,w\right)}\left(M_{y}^{r}I\right)$$

Update the neighborhood function of the winning neuron and the weights of other neurons as in Equation 12 as follows:


(12) $$\left\{ \begin{array}{c} m_{y}\left(n\right)=m_{y}\left(n-1\right)+\eta \left(n\right)TS\left(d,n\right)\left[I\left(n\right)-m_{y}\left(n-1\right)\right]\\ TS=\exp\left(\frac{d_{x,y}^{2}}{2\delta^{2}}\right)\text{,} \end{array}\right.$$

where *x* is the *x*-th neuron of the input, *y* is the *y*-th competing neuron; $m_{y}\left(n\right)$ is the weight updated at *n* iterations; $m_{y}\left(n-1\right)$ denotes the weights updated after *n*-1 iterations. The width *δ* of the neighborhood function decreases over time to satisfy Equation 13, following the decreasing function:


(13) $$\delta \left(t\right)=\delta_{0}\exp\left(-\frac{t}{\tau_{1}}\right)t=0,1\ldots$$

The numerical input of *Z* participates in the iterative process. The criteria for judging the degree of convergence are based on the error function and the criterion function, as described in Equations 14.


(14) $$Y_{C}\left(X\right)=\overset{z}{\underset{y=1}{\sum }}\overset{t_{y}}{\underset{z=1}{\sum }}i_{z}^{\left(y\right)}-K_{y}{\left(X\right)}^{2}$$

Calculate the distance from the data object in the cluster to the initial cluster center, and the obtained distance is divided into a cluster close by, as in Equation 15:


(15) $$E=\overset{Z}{\underset{Y=1}{\sum }}\underset{I\in C_{Y}}{\sum }d^{2}I_{y}-C_{w}$$

Among them, the improved k-means algorithm based on the SOM algorithm is used to cluster the data to classify the behaviors of the older adults. One activity contains the usage data of multiple devices, and then the correlation algorithm is used to analyze the linkage operation of multiple devices so that it can realize the prediction of the use of one device when another device is used, which can be used as the basis for the prediction of the next behavior activity, and the confidence level calculated by this can be used as a judgment of whether it is a user habit pattern. If the confidence level is greater than the threshold, it is considered a habit mode to store it. In a large-scale mixed data set, the user’s historical device usage information is used to analyze the correlation between multiple device control modes, and some rules with strong correlation, that is, association rules, are found. In the field of smart homes, the hidden relationship between the operations of multiple smart home devices can be excavated. In this way, the user’s “association control habits” can be realized, and multiple home devices can be integrated or linked together. Commonly used evaluation criteria for frequent item sets are support and confidence.

## Results and discussion

### Experimental dataset

The generation of the instruction set is crucial for realizing intelligent control functions. Specifically, its design must allow users to set corresponding parameters based on real-time environmental information through simple operations. The improved k-means algorithm integrates a behavior pattern classification model with time-constrained association rules to match the similarity of user-input instructions. The output is a control instruction text, thus necessitating the initial definition of smart home parameters.

Control the instruction set format and transform the historical data format according to this format for model training. The requirements for the design of the instruction set format are (1) to negotiate the communication protocol between the platform and the device, specify the language for communication, and ensure effective data exchange. (2) The structure of the instruction should conform to the communication specification of the smart home; that is, the encapsulation format of various control objects should be standardized. (3) It is necessary to clarify the target object of the instruction set, so it is necessary to cover the user’s operating habits as much as possible to simplify the operation of the smart home appliances for the older adults at home as much as possible. (4) The storage method of the instruction set should be stored according to the designed database table, and the database used is MySQL.

Devices that are often used in smart homes and their properties are explored, numbered accordingly, and categorized by numbers for attributes related to device usage habits, such as light sensors and temperature sensors. The obtained user behavior patterns are stored in the pattern instruction set.

### Dataset preparation and division

The success of the data-driven approach hinges on the quality and representativeness of the dataset. Initially, the dataset undergoes a rigorous preprocessing phase to ensure data integrity. This phase includes the application of data-cleaning techniques to address any missing values or anomalies, thereby enhancing the dataset’s reliability and quality.

Post-preprocessing, the dataset is subjected to random shuffling to guarantee an unbiased and representative split of the data. The dataset is then partitioned into three distinct subsets:

Training Set: A substantial 60% of the entire dataset is allocated to the training set. This sizable portion is crucial for enabling the model to learn from a diverse array of data points, encompassing a broad spectrum of health conditions and environmental scenarios.

Validation Set: A total of 20% of the dataset serves as the validation set. This subset plays a pivotal role in fine-tuning the model’s hyperparameters and in preventing overfitting to the training data.

Test Set: The remaining 20% of the data constitutes the test set. This subset is imperative for assessing the model’s performance in a manner that mirrors real-world smart home environments.

### Analysis of experimental results

**Figure 4:**
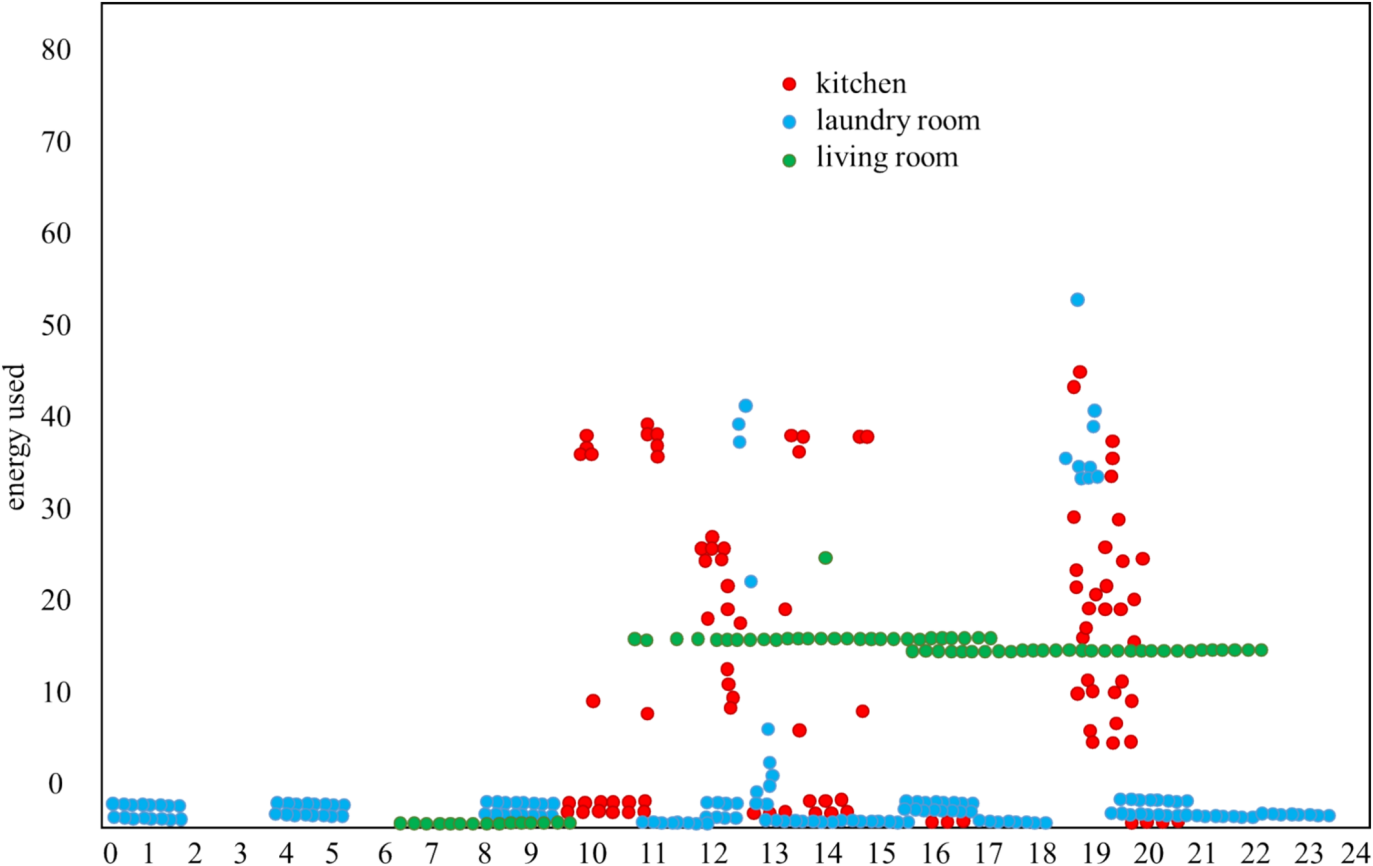
Electricity diagram of three regions.

The main content of this section involves combining the data set from UCI household electricity, including date, time, kitchen power, laundry power, water heater and air conditioner power, and other attributes. According to the characteristics of the data set, the feature vector is selected, and the electricity consumption W is collected at the same time interval from 00:00 to 24:00 in the area n. Finally, cluster analysis is performed on the data of 30 days in the three intervals. The electricity consumption data of the same device with the same electricity consumption curve will be classified into one category, and the user’s habitual characteristics of the use of a certain device can be obtained, as shown in Figure 4.

**Figure 5:**
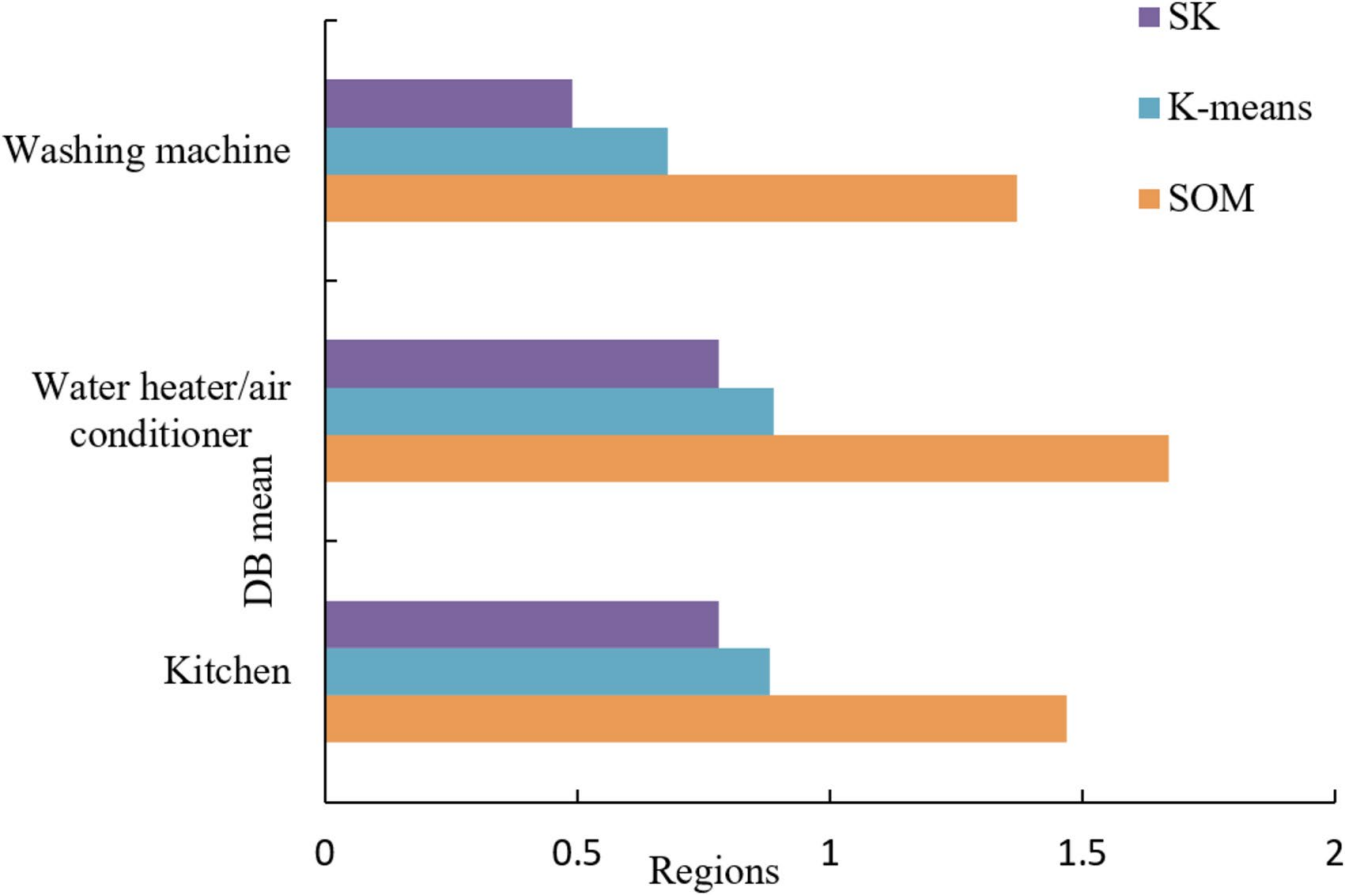
DB values of the three algorithms.

At the same time, the same evaluation index is used to evaluate the performance of SOM, k-means, and k-means algorithms fused with SOM separately in the same computing environment. The current mainstream clustering algorithm evaluation index is the average inter-class distance. The Davis-Boulding (DB) index, which represents the average within-class centroid distance, is used for evaluation. The results are shown in Figure 5.

Such a k-means algorithm fused with SOM has a good clustering effect.

Such a constructed model is trained and validated. Taking the training accuracy rate, validation accuracy rate, training loss and validation loss as comprehensive evaluation indicators, the relationship between the accuracy rate and the loss iterations is 60, the accuracy of the algorithm reaches 94%, and the training accuracy and validation accuracy are well fitted.

**Figure 6:**
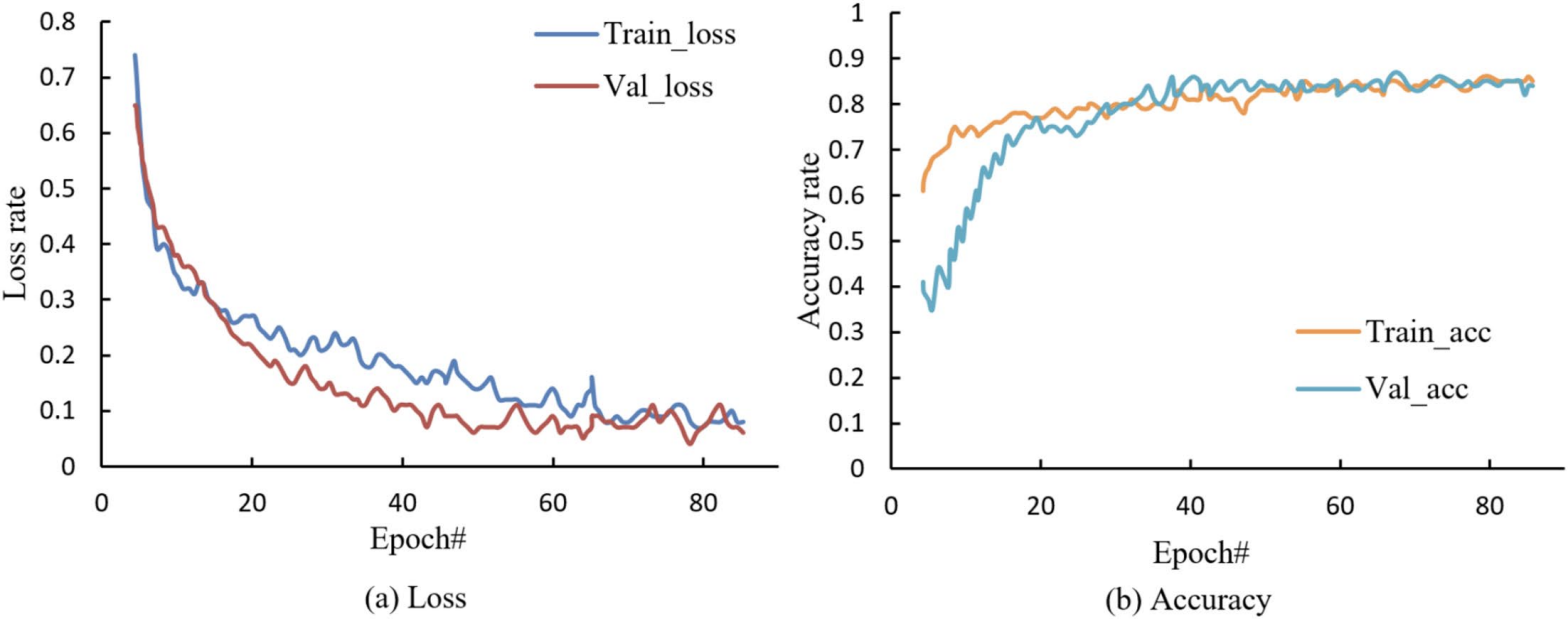
Model training effect.

**Figure 7:**
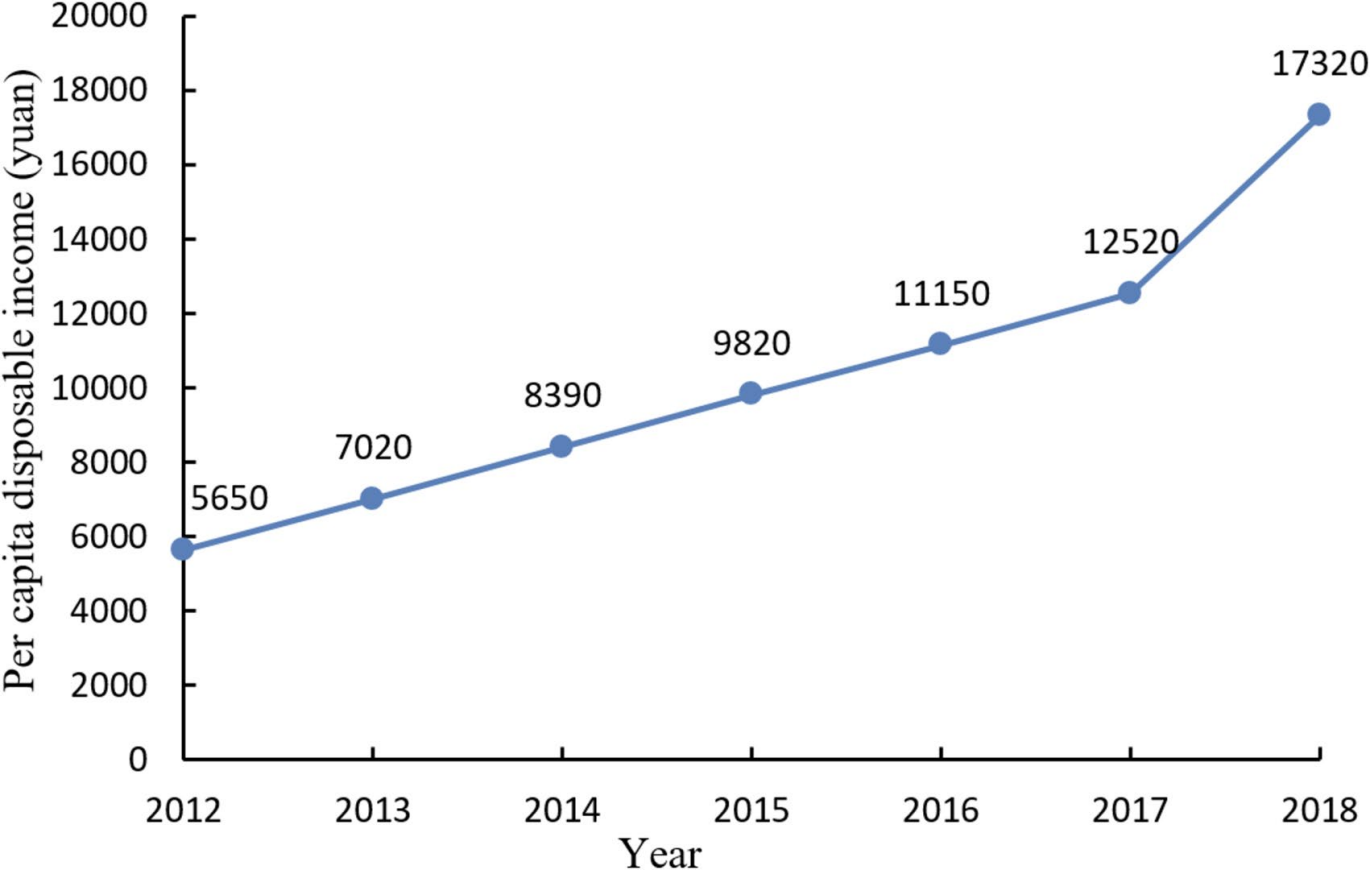
Statistical chart of *per capita* disposable income of rural residents in Qichun County from 2012 to 2018.

Figure 6 shows the model training effect. With the support of national policies and the booming domestic health industry, rural health tourism in Qichun County is gaining momentum and advancing toward maturity. The villages and towns in the county are also actively complying with this trend, leveraging their unique local resources to develop and introduce rural health tourism projects based on local conditions. Using the prediction model, the *per capita* disposable income statistics of rural residents in Qichun County from 2012 to 2018 were calculated, and the results are shown in Figure 7.

The reasons for choosing this particular time period are as follows: (1) Data availability and consistency: the 2012–2018 dataset provides a comprehensive and consistent range of data. This time period ensures the availability of complete and uninterrupted data, which is critical to the accuracy and reliability of the validation process. (2) Relevance to technological and demographic trends: 2012–2018 is a significant period of technological advancement and demographic change relevant to this study. During this period, there has been a significant growth in smart home technology and an increased focus on older adult care, which fits well with the research objectives of this study. (3) Policy and economic factors: This period also corresponds to various national policies and economic conditions that have a direct impact on the development of smart homes and older adult care services. In 2018, the operating income of rural tourism in Qichun County increased by 38.3% year-on-year, an increase of 26 percentage points compared with 2017, and the number of tourists received increased by 16% year-on-year, while rural wellness tourism accounted for 75% of rural tourism in Qichun County. and the proportion is still increasing. The development of rural health tourism in Qichun County has not only promoted the development of local tourism but also increased the *per capita* disposable income of local rural residents.

The results section demonstrates the effectiveness of the system in monitoring the health of older adults residents. Historical data from 2012 to 2018 validate the system’s ability to track health trends and provide timely interventions. This extensive data collection period ensures a comprehensive assessment of the system’s performance across various health dynamics and seasonal variations. The system’s real-time monitoring and data analysis capabilities significantly contribute to the proactive management of the older adult’s health, reducing the risk of medical emergencies and improving their overall quality of life.

## Conclusion

This study presents a comprehensive approach to older adults health monitoring and smart home assistance in sports and wellness towns through the integration of a WSN system. The results indicate that the system is effective in real-time health data collection and analysis, which are crucial for the early detection of health issues and timely intervention. The construction and optimization of the smart home system model have led to more efficient health management of physical activity for the older adults, directly improving their physical health and self-care abilities. With improved health status and reduced medical costs, the *per capita* disposable income of the older adults increases accordingly.

As income rises, the older adults will have more resources to invest in smart home systems and health management, creating a positive cycle that further promotes rural revitalization and the development of healthy towns. This study’s findings highlight the potential of technology to address the challenges faced by aging populations in rural areas while contributing to the broader goals of rural development and health equity.

## Data Availability

The original contributions presented in the study are included in the article/supplementary material, further inquiries can be directed to the corresponding author.
